# Immunohistochemical prediction of lapatinib efficacy in advanced HER2-positive breast cancer patients

**DOI:** 10.18632/oncotarget.6375

**Published:** 2015-11-24

**Authors:** Renata Duchnowska, Piotr J. Wysocki, Konstanty Korski, Bogumiła Czartoryska-Arłukowicz, Anna Niwińska, Marlena Orlikowska, Barbara Radecka, Maciej Studziński, Regina Demlova, Barbara Ziółkowska, Monika Merdalska, Łukasz Hajac, Paulina Myśliwiec, Dorota Zuziak, Sylwia Dębska-Szmich, Istvan Lang, Małgorzata Foszczyńska-Kłoda, Bożenna Karczmarek-Borowska, Anton Żawrocki, Anna Kowalczyk, Wojciech Biernat, Jacek Jassem, for the Central East European Oncology Group (CEEOG)

**Affiliations:** ^1^ Military Institute of Medicine, Oncology Department, Warsaw, Poland; ^2^ West Pomeranian Cancer Center, Szczecin, Poland; ^3^ Greater Poland Cancer Center, Poznań, Poland; ^4^ Białystok Oncology Center, Białystok, Poland; ^5^ The Maria Skłodowska-Curie Memorial Cancer Center and Institute of Oncology, Warsaw, Poland; ^6^ Warmia and Masuria Oncology Center, Olsztyn, Poland; ^7^ Opole Oncology Center, Poland; ^8^ Oncology Center, Bydgoszcz, Poland; ^9^ Masaryk Memorial Cancer Institute, Brno, Czech Republic; ^10^ Chemotherapy Department, Regional Hospital, Wrocław, Poland; ^11^ Oncology Center, Kielce, Poland; ^12^ Oncology Center, Wrocław, Poland; ^13^ Oncology Center, Zielona Góra, Poland; ^14^ Oncology Center, Bielsko-Biała, Poland; ^15^ Medical University of Łódź, Łódź, Poland; ^16^ Department of Medical Oncology, National Institute of Oncology, Budapest, Hungary; ^17^ Department of Chemotherapy, Subcarpathian Oncology Center, Rzeszów, Poland; ^18^ Medical University of Gdańsk, Gdańsk, Poland

**Keywords:** breast cancer, epidermal growth factor receptor type 2, lapatinib, mTOR, p-MAPK

## Abstract

Molecular mechanisms of lapatinib resistance in breast cancer are not well understood. The aim of this study was to correlate expression of selected proteins involved in ErbB family signaling pathways with clinical efficacy of lapatinib. Study group included 270 HER2-positive advanced breast cancer patients treated with lapatinib and capecitabine. Immunohistochemical expression of phosphorylated adenosine monophosphate-activated protein (p-AMPK), mitogen-activated protein kinase (p-MAPK), phospho (p)-p70S6K, cyclin E, phosphatase and tensin homolog were analyzed in primary breast cancer samples. The best discriminative value for progression-free survival (PFS) was established for each biomarker and subjected to multivariate analysis. At least one biomarker was determined in 199 patients. Expression of p-p70S6K was independently associated with longer (HR 0.45; 95% CI: 0.25–0.81; *p* = 0.009), and cyclin E with shorter PFS (HR 1.83; 95% CI: 1.06–3.14; *p* = 0.029). Expression of p-MAPK (HR 1.61; 95% CI 1.13–2.29; *p* = 0.009) and cyclin E (HR 2.99; 95% CI: 1.29–6.94; *p* = 0.011) was correlated with shorter, and expression of estrogen receptor (HR 0.65; 95% CI 0.43–0.98; *p* = 0.041) with longer overall survival. Expression of p-AMPK negatively impacted response to treatment (HR 3.31; 95% CI 1.48–7.44; *p* = 0.004) and disease control (HR 3.07; 95% CI 1.25–7.58; *p* = 0.015). In conclusion: the efficacy of lapatinib seems to be associated with the activity of downstream signaling pathways – AMPK/mTOR and Ras/Raf/MAPK. Further research is warranted to assess the clinical utility of these data and to determine a potential role of combining lapatinib with MAPK pathway inhibitors.

## INTRODUCTION

The introduction of trastuzumab, a monoclonal antibody directed against the epidermal growth factor 2 receptor (HER2) has led to major improvement in the treatment of patients with HER2-positive breast cancer [1–5]. The therapeutic mechanisms of trastuzumab involve both the inhibition of HER2-dependent signaling pathways and the engagement of immune responses via antibody-dependent cellular cytotoxicity [6]. Despite impressive clinical efficacy of trastuzumab, many patients are refractory to this agent or develop secondary resistance. The postulated mechanism of trastuzumab resistance include the expression of the truncated-active form of the HER2 receptor (p95HER2), the cross-talk between HER2 and insulin-like growth factor-1 receptor, the deficiency of phosphatase and tensin homologue deleted on chromosome 10 (*PTEN*) and activating mutations in the p110-alpha subunit of phosphoinositide-3-kinase (*PI3K*), and activity of *Rac1* – a Ras-like small GTPase affecting trastuzumab-mediated endocytosis of the HER2 receptor [7–19]. A small-molecule HER2 kinase inhibitor – lapatinib entered the clinical practice later than trastuzumab and has been mostly used as a second-line therapy [20]. Due to its different mode of action, the molecular resistance mechanisms of lapatinib can not be simply extrapolated from those of trastuzumab [21, 22]. The resistance to this compound may be caused by mechanisms occurring at various levels within a cancer cell: the outer/inner leaflet of the plasma membrane, cytoplasm or nucleus [14, 23–30]. Normally, activation of growth factor-associated signaling cascades is initiated at the plasma membrane in response to receptor activation (homo-, or heterodimerization) [31]. Subsequently, the signal is transmitted downstream towards the nucleus via a signaling network, which comprises multiple kinases. Signal transduction pathways in cancer cells may become activated regardless of the receptor status. Spontaneously activated signal transduction elements may be responsible for resistance to receptor-targeted therapies, since crucial pathways remain active despite receptor blockade. Hence, the activity of signal transduction molecules may potentially correlate with the resistance to lapatinib.

This study investigated the immunohistochemical (IHC) expression of selected molecules involved in the important signaling pathways associated with the family of epidermal growth factor (ErbB) receptors: phosphorylated adenosine monophosphate-activated protein alpha 1 (p-AMPK-Ser486), the mitogen-activated protein kinase (p-MAPK-T185 + Y187 + T202 + Y204), phospho (p)-p70S6K, the hypoxia-inducible factor 2 alpha (HIF2 alpha), PTEN, and cyclin E. Their status was retrospectively correlated with the clinical efficacy of lapatinib. Our aim was to shed new light on the molecular mechanisms involved in the resistance of breast cancer to lapatinib.

## RESULTS

### Patient characteristics

**Figure 1:**
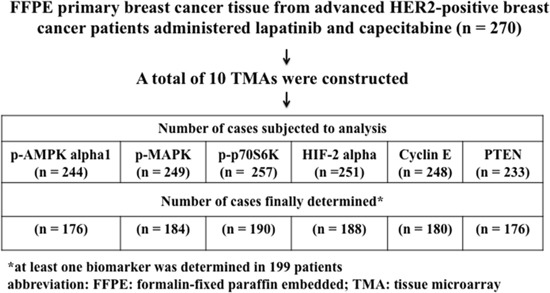
CONSORT Diagram Origin of patients analyzed for p-AMPK alpha1, p-MAPK, p-p70S6K, cyclin E, HIF2 alpha and PTEN.

Tumor samples from 270 patients were subjected to analysis, of which in 199 at least one biomarker was determined (Figure 1, Table 1). Eighty-four percent of the tumors were invasive ductal cancers (no special type), 67% were estrogen receptor (ER)-negative and 77% progesterone receptor (PR)-negative. Eleven percent of patients presented with metastatic disease at initial breast cancer diagnosis. Radical surgery was performed in 91% of patients; 98% received chemotherapy in (neo)adjuvant and/or a metastatic setting, 36% received endocrine therapy and all were administered trastuzumab in an adjuvant or a metastatic setting, usually in combination with chemotherapy. In 69% of patients, the first manifestation of progression was distant metastasis, with viscera being the most common dominant metastatic site. Forty-three percent of patients developed brain metastases during the course of their disease.

**Table 1:** Patient characteristics

Variable	*n* = 199
**Mean age at diagnosis (range)**	50 (23–81)
**Menopausal status** Premenopausal Postmenopausal	78 (43) 102 (57)
**Histology** Ductal Lobular Other Not determined Ductal and lobular	168 (84) 15 (8) 5 (3) 8 (4) 2 (1)
**Grade** 1 2 3	3 (2) 74 (46) 82 (52)
**Estrogen receptor** Negative Positive	134 (67) 65 (33)
**Progesterone receptor** Negative Positive	153 (77) 46 (23)
**Clinical stage at diagnosis** I IIA IIB IIIA IIIB IIIC IV	16 (8) 22 (12) 30 (16) 44 (23) 42 (22) 16 (8) 21 (11)
**Breast cancer surgery** No Mastectomy Breast conserving surgery	17 (9) 157 (79) 24 (12)
**Radiotherapy** No Adjuvant Definitive Palliative Combination thereof	44 (23) 70 (36) 18 (9) 24 (12) 40 (20)
**Chemotherapy** No Neoadjuvant Adjuvant For advanced disease Combination thereof	3 (2) 86 (43) 32 (16) 36 (18) 128 (64)
**Trastuzumab therapy** No Adjuvant	0 (0) 48 (24)
For advanced disease Combination thereof	136 (68) 15 (8)
**Endocrine therapy** No Neo(adjuvant) For metastatic disease Combination thereof	128 (64) 36 (18) 17 (9) 18 (9)
**Type of first progression** Local Regional Distant Combined local, regional and/or distant	20 (10) 19 (10) 138 (69) 22 (11)
**Dominant site of metastatic disease** Soft tissue Bone Visceral	34 (18) 26 (14) 125 (68)
**Brain metastases** No Yes	113 (57) 86 (43)

### Clinical outcomes

The median duration of lapatinib and capecitabine therapy was 6 months (range 0–52). In 82% of patients, treatment was terminated due to disease progression. Other reasons were toxicity (7%), patient refusal (2%), death (3%), others (5%) and unknown (1%).

The best response to a combination of lapatinib and capecitabine were CR (5%), PR (31%), stable disease (42%) and progression (16%); in the remaining 6% of patients response was unknown or not evaluated.

The duration of follow-up from breast cancer diagnosis varied from 6.7 to 242 months. The median PFS from the start of lapatinib therapy was 6.2 months (range 0–54). PFS was significantly longer in patients with response to treatment (median 10.4 months; hazard ratio [HR] 0.44, 95% confidence interval [CI] 0.35–0.56, *p* < 0.01) or disease control (median 8.1 months; HR 0.27; 95%CI 0.20–0.35; *p* < 0.01), compared to those with refractory disease (median 2.3 months).

**Figure 2:**
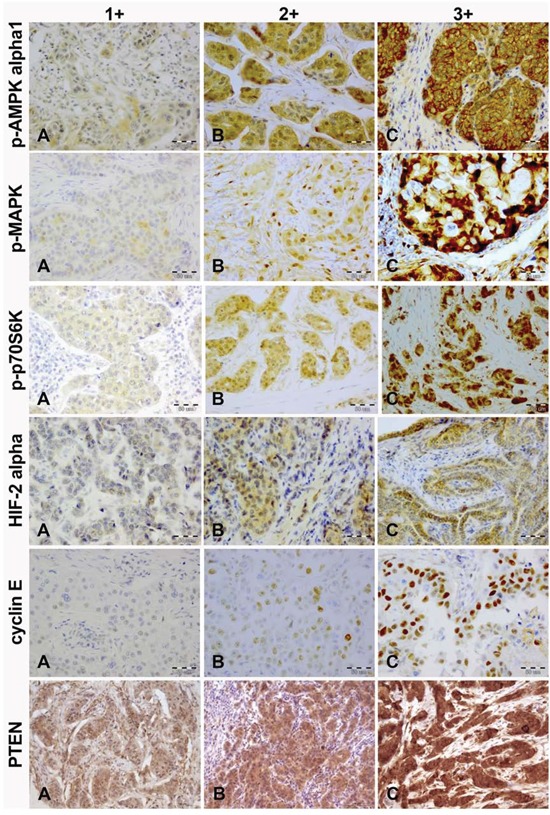
Immunohistochemical intensity scoring of p-AMPK alpha1, p-MAPK, p-p70S6K, HIF-2 alpha, cyclin E and PTEN (magnification, x20) **A.** weak; **B.** moderate; **C.** strong.

**Figure 3:**
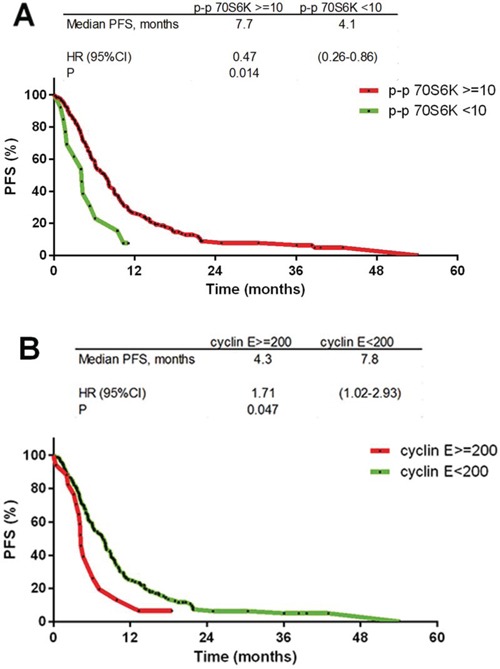
Kaplan-Meier progression free survival curves **A.** p-p70S6K ≥ 10 staining H-score (HR 0.47; *p* = 0.014); **B.** cyclin *E* ≥ 200 staining H-score (HR 1.71; *p* = 0.05).

The status of p-AMPK alpha1, p-MAPK, p-p70S6K, HIF-2 alpha, cyclin E and PTEN was determined in 176, 184, 190, 188, 180 and 176 cases, respectively (CONSORT Diagram, Figure 1). The immunostained sections of all studied proteins are shown on Figure 2. In all cases staining was heterogeneous. For cyclin E the staining was nuclear, for HIF-2 cytoplasmic and for p-AMPK alpha1, p-MAPK, p-p70S6K, and PTEN nuclear and cytoplasmic. Two of the examined biomarkers: p-p70S6K and cyclin E proved predictive for PFS, with the best discriminating H-scores of 10 and 200, respectively. The expression of p-p70S6K (HR 0.47; 95%CI 0.26–0.86; *p* = 0.014) was associated with longer, and the expression of cyclin E (HR 1.71; 95%CI 1.00–2.93; *p* = 0.05) with shorter PFS. The predictive value of these two biomarkers was confirmed in the multivariate analysis (HR 0.45; 95% CI 0.25–0.81; *p* = 0.009 and 1.83; 95%CI 1.06–3.14; *p* = 0.029, respectively; Figure 3A–3B and Table 2).

**Table 2:** Hazard ratios for progression free (PFS) and overall survival (OS): univariate and multivariate analyses

Univariate analysis		
Variable	PFS HR (95%CI); *p*	OS HR (95%CI); *p*
p-p70S6K Cyclin E p-MAPK HIF-2 alpha PTEN p-AMPK Type of first progression* ER negative vs positive	0.47 (0.26–0.86); 0.014 1.71 (1.00–2.93); 0.05 0.89 (0.60–1.29); 0.51 1.23 (0.60–2.51); 0.57 1.04 (0.66–1.62); 0.88 0.94 (0.23–3.79); 0.92 1.30 (0.91–1.86); 0.15 0.95 (0.76–1.19); 0.65	0.82 (0.43–1.56); 0.545 2.86 (1.23–6.66); 0.015 1.68 (1.18–2.40); 0.007 0.77 (0.39–1.52); 0.45 1.08 (0.69–1.70); 0.728 0.96 (0.31–3.03); 0.95 3.39 (1.38–8.28); 0.008 0.60 (0.39–0.92); 0.033
**Multivariate analysis**		
**Variable**	**PFS HR (95%CI); *p***	**OS HR (95% CI); *p***
p-p70S6K Cyclin E p-MAPK Type of first progression* ER negative vs positive	0.45 (0.25–0.81); 0.009 1.83 (1.06–3.14); 0.029 NC NC NC	NC 2.99 (1.29–6.94); 0.011 1.61 (1.13–2.29); 0.009 1.87 (1.02–3.44); 0.044 0.65 (0.4–0.98); 0.041

PFS: progression free survival; OS: overall survival; ER: estrogen receptor;

* regional vs. local; NC: not calculated (*P* > 0.1 in univariate analysis and not included in the stepwise and Cox regression multivariate analysis

**Figure 4:**
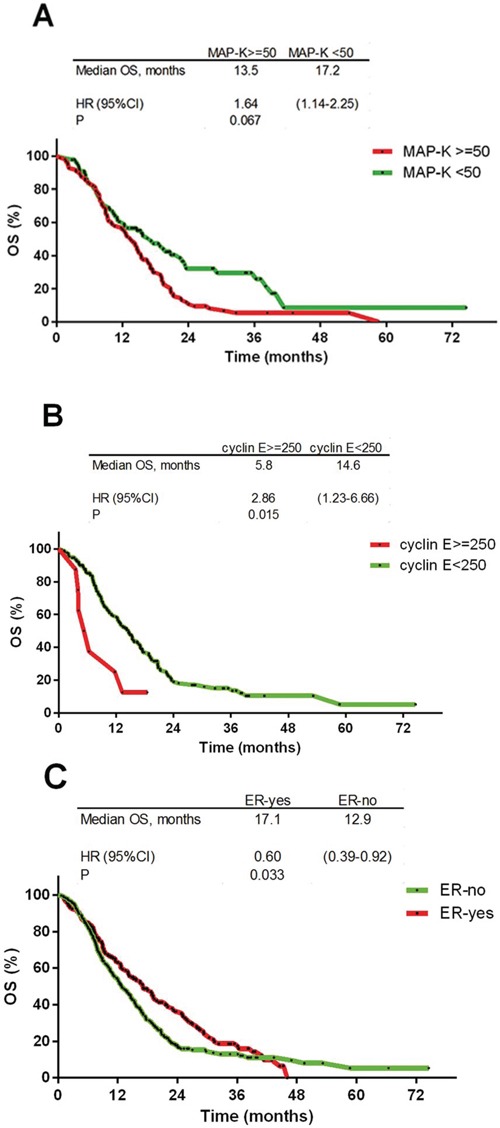
Kaplan-Meier overall survival curves **A.** p-MAPK ≥ 50 staining H-score (HR 1.68; *p* = 0.0007); **B.** cyclin *E* ≥ 250 staining H-score (HR 2.86; *p* = 0.0015); **C.** estrogen receptor: positive vs. negative (HR 0.60; *p* = 0.033).

Negative prognostic factors for OS included the expression of p-MAPK (HR 1.68; 95%CI 1.18–2.40; *p* = 0.007) and cyclin E (HR 2.86; 95%CI 1.23–6.66; *p* = 0.015; Figure 4A–4B and Table 2), in addition to regional vs. local type of first progression (HR 3.39; 95%CI 1.38–8.28; *p* = 0.008), whereas ERα expression positively impacted OS (HR 0.60; 95%CI 0.39–0.92; *p* = 0.033; Figure 4C and Table 2). The significance of these biomarkers was confirmed in the multivariate analysis (Table 2).

The expression of p-AMPK alpha1 negatively impacted response to treatment (HR 3.31; 95%CI 1.48–7.44; *p* = 0.004) and disease control (HR 3.07; 95%CI 1.25–7.58; *p* = 0.015) in the multivariate analysis.

A subset analysis considering ER status showed that p-MAPK expression in the ER-positive cohort was associated with significantly shorter PFS (HR 3.14; 95%CI 1.59–6.20; *p* = 0.001) and OS (HR 2.53; 95%CI 1.05–6.11; *p* = 0.038), whereas no such correlation was seen in the ER-negative cohort (Table 3). Another biomarker with adverse impact in the ER-positive cohort was HIF-2 alpha (HR for OS 3.38; 95%CI 1.13–10.08; *p* = 0.029). In turn, the expression p-p70S6K in the ER-positive cohort was associated with longer PFS (HR 0.22; 95%CI 0.06–0.75; *p* = 0.016; Table 3). The expression of cyclin E was more common in the ER-negative cohort (*p* = 0.003) and in this subset associated with shorter PFS (HR 1.78; 95%CI 1.02–3.09; *p* = 0.041) and OS (HR 2.38; 95%CI 1.09–5.18; *p* = 0.029). No such impact of cyclin E was found in the ER-positive subgroup. The significance of these biomarkers was confirmed in the multivariate analysis.

**Table 3:** Hazard ratios for progression free (PFS) and overall survival (OS): a subset univariate and multivariate analyses considering ER status

Univariate analysis			
Variable	N	PFS HR (95%CI); *p*	OS HR (95%CI); *p*
**ER-positive**			
p-p70S6K Cyclin E p-MAPK HIF-2 alpha PTEN p-AMPK	61 54 57 58 60 54	0.22 (0.06–0.75); 0.016 1.71 (0.53–5.55); 0.37 3.14 (1.59–6.20); 0.001 1.86 (0.58–6.02); 0.299 0.88 (0.41–1.88); 0.733 0.54 (0.17–1.78); 0.313	0.45 (0.18–1.09); 0.077 1.21 (0.29–5.12); 0.795 2.53 (1.05–6.11); 0.038 3.38 (1.13–10.08); 0.029 1.67 (0.70–4.01); 0.251 0.41 (0.12–1.35); 0.142
**ER-negative**			
p-p70S6K Cyclin E p-MAPK HIF-2 alpha PTEN p-AMPK	129 126 127 130 116 122	0.66 (0.31–1.44); 0.299 1.78 (1.02–3.09); 0.041 0.89 (0.59–1.41); 0.611 0.87 (0.35–2.15); 0.768 1.14 (0.65–1.99); 0.64 1.80 (0.44–7.32); 0.41	0.65 (0.31–1.34); 0.24 2.38 (1.09–5.18); 0.029 0.87 (0.54–1.38); 0.548 0.73 (0.30–1.80); 0.494 0.79 (0.46–1.34); 0.375 1.17 (0.37–3.71); 0.786
**Multivariate analysis**			
**Variable N**	**N**	**PFS HR (95%CI); *p***	**HR (95%CI); *p***
**ER-positive**			
p-p70S6K p-MAPK HIF-2 alpha	61 57 58	0.10 (0.02–0.38); 0.001 4.48 (1.97–10.18); 0.001 1.51 (0.73–3.13); 0.263	0.23 (0.06–0.81); 0.023 3.91 (1.71–8.90); 0.001 4.74 (1.49–15.07); 0.008
**ER-negative**			
Cyclin E	126	1.78 (1.02–3.09); 0.041	2.38 (1.09–5.18); 0.029

PFS: progression free survival; OS: overall survival; ER: estrogen receptor; N: number of cases

## DISCUSSION

Despite spectacular progress in the treatment of HER2-positive breast cancer, overcoming primary and acquired resistance to anti-HER2 agents remains a critical challenge [32, 33]. In contrast to trastuzumab, the anti-tumor activity of lapatinib is based solely on the intracellular inhibition of cell-signaling by competing with ATP for the ATP-binding domain in the cytoplasmic tail of the tyrosine kinase receptor – mostly HER2 and EGFR [34, 35]. Accordingly, the postulated mechanisms underlying lapatinib resistance differs from those reported for trastuzumab. Previous studies have shown up-regulated ER-associated signaling genes, including FOXO3a and caveolin-1, or Akt pathway transcripts (AKT1, MAPK9, HSPCA, IRAK1, CCND1) in lapatinib resistant cells [36]. Other factors contributing to lapatinib resistance include dominant activating mutations in *PIK3CA*, *E545K* and *H1047R* or overexpression of AXL, a membrane-bound tyrosine kinase receptor with resulting crosstalk between HER, AXL and ER receptor pathways [37]. There are two major signaling pathways controlled by receptors belonging to the ErbB-receptors family – Ras/Raf/MAPK, regulating cell division and proliferation, and PI3K/Akt/mTOR, regulating cell growth and survival [38]. Hence, particular impairments in these pathways result in improper activation of signaling cascades and may influence the clinical efficacy of lapatinib [39, 40].

Our study suggests that another key element involved in regulation of mTOR1 complex – phosporylated AMPK alpha 1 protein kinase – may negatively impact response to lapatinib. AMPK acts as a crucial regulator of cell growth, proliferation and autophagy [41–42]. Intensive cellular energy-consuming processes, such as glucose deprivation, hypoxia, oxidative stress, hyperosmotic stress, or tissue ischemia, result in increased concentration of AMP, which leads to AMPK activation. Subsequently, activated AMPK, via phosphorylation of raptor or TSC2, inhibits activity of mTORC1, leading to general blockade of cellular anabolic processes and simultaneously activating catabolic processes [43]. The direct phosphorylation of raptor by AMPK leads to mTORC1 disruption and cell cycle arrest induced by energy stress [44–47]. Alteration of mTOR signaling networks, which is a common phenomenon in human cancers, may result from impairment of upstream regulatory mechanisms [48].

We showed that p-70S6K phosphorylation, reflecting the PI3K/AKT/mTOR pathway activity, was associated with improved PFS in lapatinib-treated patients, particularly in those with ER-positive tumors. Phosphorylation of p-70S6K depends solely on the activation of mTORC1, whereas p-70S6K exerts a negative feedback loop that inhibits PI3K/Akt via IRS-1 [47]. It is possible, that the favorable impact of p70S6K is associated with its inhibitory activity against various (not only ErbB-family members) membrane receptor complexes. However, a genuine predictive value of p-70S6K for lapatinib would necessitate testing this biomarker also in lapatinib-untreated patients, to exclude its possible favorable prognostic impact shown previously in early ERα-positive breast cancer [49]. In our study a member of Ras/Raf/MAPK pathway – p-MAPK, appeared to be a negative prognostic factor, mainly in patients with ER-positive tumors. This may indicate that in advanced HER2-positive breast cancer patients treated with lapatinib, phosphorylation of p-70S6K reflects significant activity of the Ras/Raf/MAPK pathway, particularly when accompanied by p-AMPK up-regulation. However, since p-MAPK is a distant downstream element of the Ras/Raf/MAPK signaling pathway, its activity may result from crosstalk with various distinct signaling pathways. This observation may also suggest a potential role of combining lapatinib with MAPK pathway inhibitors. Previous studies have shown that, unlike trastuzumab, lapatinib affects cell cycle kinetics through Ras/MAPK, and had less effect on cell survival [14].

Our results suggest that the potential anti-tumor role of AMPK activators, such as metformin, may be limited in lapatinib-treated patients and requires further research [50]. A phase I trial evaluating a combination of lapatinib with metformin or sirolimus (mTOR inhibitor) in advanced cancer patients is currently ongoing (www.clinicaltrials.gov; NCT01087983). Similarly to previous studies [14, 22], we have not found a correlation between the PTEN status and clinical efficacy of lapatinib. Indeed, the activation of the PI3K/Akt/mTOR pathway resulting from mutations of *PIK3CA* or loss/mutations of *PTEN* has been attributed to the development of resistance to trastuzumab [7–12, 14] but not to lapatinib [14, 22].

As expected, in this series a high expression of cell cycle-regulating protein – cyclin E was more common in the ER-negative tumors, and in this subset was associated with apparently shorter PFS and OS. This biomarker was earlier shown to confer a poor clinical outcome in breast cancer [51]. Recent study demonstrated that cyclin E levels decrease upon HER2 down-regulation and HER2 inhibition, suggesting that HER2 regulates cyclin E function [52]. Finally, in a small group of HER2-positive breast cancer patients treated with trastuzumab-based therapy, cyclin E amplification or overexpression was associated with significantly impaired clinical outcomes [52]. Taken together, these data indicate that cyclin E may represent another potential therapeutic target in overcoming lapatinib resistance.

In our study expression of HIF-2 alpha was associated with poor OS in the subset of ER-positive tumors. HIF-2 alpha is a key regulatory factor in tumor growth and its adverse prognostic impact has been previously reported [53, 54].

Not surprisingly, our study showed impaired survival in patients with ER-negative tumors. A negative prognostic impact of ER-negativity in HER2-positive breast cancer patients, was earlier reported by other authors [55, 56]. Indeed, the clinical behavior (including tumor kinetics and sites of recurrence) of ER-positive/HER2 positive subtype (HER2-positive luminal B breast cancer) differs from that of ER-negative HER2 enriched subtype [55–58].

A recent study suggested that the clinical benefit of first-line trastuzumab in advanced breast cancer may be predictive for the efficacy of second- and later lines of anti-HER2 therapies [59]. As our series included patients exposed to trastuzumab in both adjuvant and metastatic setting, we were unable to include this variable in the analysis.

In conclusion, our study suggests that the clinical efficacy of lapatinib may be associated with the activity of downstream signaling pathways – AMPK/mTOR and Ras/Raf/MAPK. These data may indicate a potential role of combining lapatinib with MAPK pathway inhibitors and justify further research on combinations of lapatinib with mTOR inhibitors, such as everolimus. We are aware of several limitations of this study. First, our investigations included only downstream signaling pathways, and not the underlying molecular alterations. Second, although our series was homogeneous, i.e. all patients were treated with lapatinib and capecitabine, a nonrandomized study design did not allow to test the predictive value of particular markers. Notably, a proportion of samples was excluded from analysis due to analytical problems, and there was no lapatinib-untreated control group. Finally, this analysis used material obtained from the primary tumor, whereas several studies showed phenotypic instability of metastatic sites, particularly in relation to hormone receptors [60, 61]. The question of whether biomarkers analyzed in this study are also a subject of such conversions, and whether this impacts response to lapatinib, remains to be answered. Hence, these results should be considered preliminary and only hypothesis generating. Further investigations are warranted to verify the clinical utility of our findings.

## MATERIALS AND METHODS

### Patients

This study was approved by the Institutional Review Board of the coordinating center (the Military Institute of Medicine in Warsaw, Poland). An initial study population included HER2-positive advanced breast cancer patients treated in 31 oncology centers in Poland, Hungary, Czech Republic, Lithuania and Romania between 2004 and 2013. The patients should have received a combination of lapatinib at an initial dose of 1250 mg per day continuously and capecitabine at a dose of 2000 mg/m^2^ of body-surface area on days 1 through to 14 of a 21-day cycle for at least 6 weeks. Patients must have earlier received trastuzumab and 1–3 lines of chemotherapy for advanced disease. Other eligibility criteria included age above 18 years, no previous or concomitant malignant disease except for basal cell carcinoma of the skin, tumor lesions evaluable for therapeutic response to lapatinib and the availability of formalin-fixed, paraffin embedded (FFPE) tumor tissue specimens for analysis. The following information was extracted from the medical records: the date of breast cancer diagnosis, previous local and systemic therapy, the date and type of the first progression (local, regional, distant), the dominant site of metastatic disease (soft tissue, bone, viscera), the date of brain metastasis diagnosis, the dates on which lapatinib and capecitabine were administered, the date of the first progression while on lapatinib and capecitabine therapy, and the date of death or the last follow-up visit. For tumors involving more than one category, the dominant site of distant disease was classified by the category associated with the worst prognosis, irrespective of the extent of involvement, in the following order of increasing gravity: soft tissue, bones, and viscera. Due to the retrospective nature of this study, tumor staging was performed using the American Joint Committee on Cancer/the Union for International Cancer Control classification from 1997. Follow-up information was extracted from medical records and tumor registries. All data were coded to secure full protection of personal information. Alive patients had to provide written informed consent for the use of their archival tumor samples for analysis, according to regulations in particular countries.

### Pathology

The starting material from each patient was an archival formalin-fixed, paraffin embedded (FFPE) specimen(s) from the primary breast cancer obtained at surgery or by tissue biopsy. A pre-cut section of each tumor, stained with hematoxylin and eosin, was reviewed by two board-certified pathologists (WB and KK) to confirm breast cancer diagnosis and determine whether a sufficient invasive breast cancer component was present (1 cm^2^ invasive tissue; ≥ 30% tumor cells). In each case, assuming potential intratumoral heterogeneity, 2 tissue cores (1.5 mm in diameter) were punched out from the FFPE tissue blocks containing primary breast cancer (“donor”) and transferred into a “recipient” paraffin block using Manual Tissue MicroArrayer (MTA I, Beecher Instrument Inc.) A total of 10 tissue microarrays (TMAs) were constructed.

### Immunohistochemistry (IHC) staining

IHC analysis was performed in tumor tissue in accordance with standard protocols, on 5 μm histological slides derived from the TMA blocks. The tumor-associated stromal cells were not analyzed. The staining was performed according to manufactures' protocols with the use of the following antibodies: p-AMPK alpha1 (ab39400 rabbit polyclonal, Abcam, Cambridge, UK; dilution 1:300), p-MAPK (ab50011 mouse monoclonal, Abcam, Cambridge, UK; dilution 1:100), p-p70S6K (ab32359 rabbit monoclonal, Abcam, Cambridge, UK; dilution 1:25), HIF2alpha (ab20654 rabbit monoclonal, Abcam, Cambridge, UK; dilution 1:250), cyclin E (HE12 mouse monoclonal, Thermo Sci, Waltham, MA, USA; dilution 1:50) and PTEN (6H2.1 mouse monoclonal, DAKO Denmark; dilution 1:100). Positive controls were used according to manufacturer's recommendations and negative controls included standard staining procedures with the omitting of the primary antibody step. TMA sections were deparaffinized in xylene and rehydrated through graded alcohols. Antigen retrieval procedure was performed using Target Retrieval Solution, with pH depending on monoclonal antibody in electric pressure cooker, followed by 20 min cooling before further immunostaining. Endogenous reactivity was blocked with normal goat serum. Following the preliminary stages, incubation with the primary antibody was carried out for 30 minutes. The binding of the monoclonal antibody was detected with biotin-labeled goat anti-mouse or anti-rabbit immunoglobulin G (IgG) and horseradish peroxidase-labeled avidin – biotin complex. IHC stains were scored manually according to staining intensity (0 – negative, 1 – weak, 2 – moderate, 3 – strong) and the percentage of positive tumor cells. Each tissue core was assessed separately and the core with the highest staining intensity was considered representative for the particular case. To accurately describe the extent of immunohistochemical staining of a tumor and to potentially increase the predictive information, expression of particular biomarkers was assessed using the staining H-score. The H-score was calculated for each biomarker by the formula: 3 × percentage of strong cellular (cytoplasmic or nuclear wherever appropriate) staining plus 2 × percentage of moderate cellular staining plus percentage of weak cellular staining, giving a range of 0 to 300. The cutoff values for each biomarker were optimized using Cox regression model to maximize the hazard ratio (HR) between patients with expression levels above vs. below the cutoff.

### Statistical analysis

All statistical analyses were performed using STATA software version 11. Statistical significance was defined as *p* < 0.05. Categorical variables were compared using Pearson's chi-squared test (χ^2^) and Spearman's R rang.

The primary endpoint was progression free survival (PFS), defined as the time from the date of the lapatinib start to the date of the disease progression or death, whichever occurred first. The secondary endpoints were an objective response, defined as a complete response (CR) or a partial response (PR) and disease control, defined as CR, PR and stable disease combined, determined according to Response Evaluation Criteria in Solid Tumors (RECIST) v 1.0 criteria. Survival curves were plotted using the Kaplan-Meier method, starting from the first day of lapatinib therapy to the date of death or the last follow-up. Univariate analyses were performed with a log-rank test, Wilcoxon test and Cox proportional hazard and logistic regression. Multivariate analysis used a stepwise forward selection of univariate model with *p* ≤ 0.10.

## References

[1] Slamon DJ, Leyland-Jones B, Shak S, Fuchs H, Paton V, Bajamonde A, Fleming T, Eiermann W, Wolter J, Pegram M, Baselga J, Norton L (2001). Use of chemotherapy plus a monoclonal antibody against HER2 for metastatic breast cancer that overexpresses HER2. N Engl J Med.

[2] Romond EH, Perez EA, Bryant J, Suman VJ, Geyer CE, Davidson NE, Tan-Chiu E, Martino S, Paik S, Kaufman PA, Swain SM, Pisansky TM, Fehrenbacher L (2005). Trastuzumab plus adjuvant chemotherapy for operable HER2-positive breast cancer. N Engl J Med.

[3] Piccart-Gebhart MJ, Procter M, Leyland-Jones B, Goldhirsch A, Untch M, Smith I, Gianni L, Baselga J, Bell R, Jackisch C, Cameron D, Dowsett M, Barrios CH (2005). Trastuzumab after adjuvant chemotherapy in HER2-positive breast cancer. N Engl J Med.

[4] Joensuu H, Kellokumpu-Lehtinen PL, Bono P, Alanko T, Kataja V, Asola R, Utriainen T, Kokko R, Hemminki A, Tarkkanen M, Turpeenniemi-Hujanen T, Jyrkkiö S, Flander M (2006). Adjuvant docetaxel or vinorelbine with or without trastuzumab for breast cancer. N Engl J Med.

[5] Slamon D, Eiermann W, Robert N, Pienkowski T, Martin M, Press M, Mackey J, Glaspy J, Chan A, Pawlicki M, Pinter T, Valero V, Liu MC (2011). Adjuvant trastuzumab in HER2-positive breast cancer. N Engl J Med.

[6] Petricevic B, Laengle J, Singer J, Sachet M, Fazekas J, Steger G, Bartsch R, Jensen-Jarolim E, Bergmann M (2013). Trastuzumab mediates antibody-dependent cell-mediated cytotoxicity and phagocytosis to the same extent in both adjuvant and metastatic HER2/neu breast cancer patients. J Transl Med.

[7] Spector NL, Blackwell KL (2009). Understanding the mechanisms behind trastuzumab therapy for human epidermal growth factor receptor 2-positive breast cancer. J Clin Oncol.

[8] Nagata Y, Lan KH, Zhou X, Tan M, Esteva FJ, Sahin AA, Klos KS, Li P, Monia BP, Nguyen NT, Hortobagyi GN, Hung MC, Yu D (2004). PTEN activation contributes to tumor inhibition by trastuzumab, and loss of PTEN predicts trastuzumab resistance in patients. Cancer Cell.

[9] Wang Y, Liu Y, Du Y, Yin W, Lu J (2013). The predictive role of phosphatase and tensin homolog (PTEN) loss, phosphoinositol-3 (PI3) kinase (PIK3CA) mutation, and PI3K pathway activation in sensitivity to trastuzumab in HER2-positive breast cancer: a meta-analysis. Curr Med Res Opin.

[10] Duman BB, Sahin B, Acikalin A, Ergin M, Zorludemir S (2013). PTEN, Akt, MAPK, p53 and p95 expression to predict trastuzumab resistance in HER2 positive breast cancer. J BUON.

[11] Gori S, Sidoni A, Colozza M, Ferri I, Mameli MG, Fenocchio D, Stocchi L, Foglietta J, Ludovini V, Minenza E, De Angelis V, Crinò L (2009). EGFR, pMAPK, pAkt and PTEN status by immunohistochemistry: correlation with clinical outcome in HER2-positive metastatic breast cancer patients treated with trastuzumab. Ann Oncol.

[12] Esteva FJ, Guo H, Zhang S, Santa-Maria C, Stone S, Lanchbury JS, Sahin AA, Hortobagyi GN, Yu D (2010). PTEN, PIK3CA, p-AKT, and p-p70S6K status: association with trastuzumab response and survival in patients with HER2-positive metastatic breast cancer. Am J Pathol.

[13] Wang L, Zhang Q, Zhang J, Sun S, Guo H, Jia Z, Wang B, Shao Z, Wang Z, Hu X (2011). PI3K pathway activation results in low efficacy of both trastuzumab and lapatinib. BMC Cancer.

[14] Dave B, Migliaccio I, Gutierrez MC, Wu MF, Chamness GC, Wong H, Narasanna A, Chakrabarty A, Hilsenbeck SG, Huang J, Rimawi M, Schiff R, Arteaga C (2011). Loss of phosphatase and tensin homolog or phosphoinositol-3 kinase activation and response to trastuzumab or lapatinib in human epidermal growth factor receptor 2-overexpressing locally advanced breast cancers. J Clin Oncol.

[15] Raina D, Uchida Y, Kharbanda A, Rajabi H, Panchamoorthy G, Jin C, Kharbanda S, Scaltriti M, Baselga J, Kufe D (2014). Targeting the MUC1-C oncoprotein downregulates HER2 activation and abrogates trastuzumab resistance in breast cancer cells. Oncogene.

[16] Nahta R, Yuan LX, Zhang B, Kobayashi R, Esteva FJ (2005). Insulin-like growth factor-I receptor/human epidermal growth factor receptor 2 heterodimerization contributes to trastuzumab resistance of breast cancer cells. Cancer Res.

[17] Lu Y, Zi X, Zhao Y, Mascarenhas D, Pollak M (2001). Insulin-like growth factor-I receptor signaling and resistance to trastuzumab (Herceptin). J Natl Cancer Inst.

[18] Zhang S, Huang WC, Li P, Guo H, Poh SB, Brady SW, Xiong Y, Tseng LM, Li SH, Ding Z, Sahin AA, Esteva FJ, Hortobagyi GN, Yu D (2011). Combating trastuzumab resistance by targeting SRC, a common node downstream of multiple resistance pathways. Nat Med.

[19] Scaltriti M, Eichhorn PJ, Cortés J, Prudkin L, Aura C, Jiménez J, Chandarlapaty S, Serra V, Prat A, Ibrahim YH, Guzmán M, Gili M, Rodríguez O (2011). Cyclin E amplification/overexpression is a mechanism of trastuzumab resistance in HER2+ breast cancer patients. Proc Natl Acad Sci U S A.

[20] Geyer CE, Forster J, Lindquist D, Chan S, Romieu CG, Pienkowski T, Jagiello-Gruszfeld A, Crown J, Chan A, Kaufman B, Skarlos D, Campone M, Davidson N (2006). Lapatinib plus capecitabine for HER2-positive advanced breast cancer. N Engl J Med.

[21] Xia W, Mullin RJ, Keith BR, Liu LH, Ma H, Rusnak DW, Owens G, Alligood KJ, Spector NL (2002). Anti-tumor activity of GW572016: a dual tyrosine kinase inhibitor blocks EGF activation of EGFR/erbB2 and downstream Erk1/2 and AKT pathways. Oncogene.

[22] Xia W, Husain I, Liu L, Bacus S, Saini S, Spohn J, Pry K, Westlund R, Stein SH, Spector NL (2007). Lapatinib antitumor activity is not dependent upon phosphatase and tensin homologue deleted on chromosome 10 in ErbB2-overexpressing breast cancers. Cancer Res.

[23] Wang YC, Morrison G, Gillihan R, Guo J, Ward RM, Fu X, Botero MF, Healy NA, Hilsenbeck SG, Phillips GL, Chamness GC, Rimawi MF, Osborne CK (2011). Different mechanisms for resistance to trastuzumab versus lapatinib in HER2-positive breast cancers-role of estrogen receptor and HER2 reactivation. Breast Cancer Res.

[24] Wetterskog D, Shiu KK, Chong I, Meijer T, Mackay A, Lambros M, Cunningham D, Reis-Filho JS, Lord CJ, Ashworth A (2014). Identification of novel determinants of resistance to lapatinib in ERBB2-amplified cancers. Oncogene.

[25] Brady SW, Zhang J, Seok D, Wang H, Yu D (2014). Enhanced PI3K p110α signaling confers acquired lapatinib resistance that can be effectively reversed by a p110α-selective PI3K inhibitor. Mol Cancer Ther.

[26] De Luca A, D'Alessio A, Gallo M, Maiello MR, Bode AM, Normanno N (2014). Src and CXCR4 are involved in the invasiveness of breast cancer cells with acquired resistance to lapatinib. Cell Cycle.

[27] Sato Y, Yashiro M, Takakura N (2013). Heregulin induces resistance to lapatinib-mediated growth inhibition of HER2-amplified cancer cells. Cancer Sci.

[28] Wang Q, Quan H, Zhao J, Xie C, Wang L, Lou L (2013). RON confers lapatinib resistance in HER2-positive breast cancer cells. Cancer Lett.

[29] Johnston S, Trudeau M, Kaufman B, Boussen H, Blackwell K, LoRusso P, Lombardi DP, Ben Ahmed S, Citrin DL, DeSilvio ML, Harris J, Westlund RE, Salazar V (2008). Phase II study of predictive biomarker profiles for response targeting human epidermal growth factor receptor 2 (HER-2) in advanced inflammatory breast cancer with lapatinib monotherapy. J Clin Oncol.

[30] Vazquez-Martin A, Oliveras-Ferraros C, Colomer R, Brunet J, Menendez JA (2008). Low-scale phosphoproteome analyses identify the mTOR effector p70 S6 kinase 1 as a specific biomarker of the dual-HER1/HER2 tyrosine kinase inhibitor lapatinib (Tykerb) in human breast carcinoma cells. Ann Oncol.

[31] Yarden Y (2001). Biology of HER2 and its importance in breast cancer. Oncology.

[32] Puglisi F, Minisini AM, De Angelis C, Arpino G (2012). Overcoming treatment resistance in HER2-positive breast cancer: potential strategies. Drugs.

[33] Arteaga CL, Sliwkowski MX, Osborne CK, Perez EA, Puglisi F, Gianni L (2011). Treatment of HER2-positive breast cancer: current status and future perspectives. Nat Rev Clin Oncol.

[34] Rusnak DW, Affleck K, Cockerill SG, Stubberfield C, Harris R, Page M, Smith KJ, Guntrip SB, Carter MC, Shaw RJ, Jowett A, Stables J, Topley P (2001). The characterization of novel, dual ErbB-2/EGFR, tyrosine kinase inhibitors: potential therapy for cancer. Cancer Res.

[35] Rusnak DW, Lackey K, Affleck K, Wood ER, Alligood KJ, Rhodes N, Keith BR, Murray DM, Knight WB, Mullin RJ, Gilmer TM (2001). The effects of the novel, reversible epidermal growth factor receptor/ErbB-2 tyrosine kinase inhibitor, GW2016, on the growth of human normal and tumor-derived cell lines *in vitro* and *in vivo*. Mol Cancer Ther.

[36] Hegde PS, Rusnak D, Bertiaux M, Alligood K, Strum J, Gagnon R, Gilmer TM (2007). Delineation of molecular mechanisms of sensitivity to lapatinib in breast cancer cell lines using global gene expression profiles. Mol Cancer Ther.

[37] Liu L, Greger J, Shi H, Liu Y, Greshock J, Annan R, Halsey W, Sathe GM, Martin AM, Gilmer TM (2009). Novel mechanism of lapatinib resistance in HER2-positive breast tumor cells: activation of AXL. Cancer Res.

[38] Steelman LS, Chappell WH, Abrams SL, Kempf RC, Long J, Laidler P, Mijatovic S, Maksimovic-Ivanic D, Stivala F, Mazzarino MC, Donia M, Fagone P, Malaponte G (2011). Roles of the Raf/MEK/ERK and PI3K/PTEN/Akt/mTOR pathways in controlling growth and sensitivity to therapy-implications for cancer and aging. Aging (Albany NY).

[39] McCubrey JA, Steelman LS, Chappell WH, Abrams SL, Franklin RA, Montalto G, Cervello M, Libra M, Candido S, Malaponte G, Mazzarino MC, Fagone P, Nicoletti F (2012). Ras/Raf/MEK/ERK and PI3K/PTEN/Akt/mTOR cascade inhibitors: how mutations can result in therapy resistance and how to overcome resistance. Oncotarget.

[40] Lauring J, Park BH, Wolff AC (2013). The phosphoinositide-3-kinase-Akt-mTOR pathway as a therapeutic target in breast cancer. J Natl Compr Canc Netw.

[41] Hardie DG, Scott JW, Pan DA, Hudson ER (2003). Management of cellular energy by the AMP-activated protein kinase system. FEBS Lett.

[42] Hardie DG, Carling D (1997). The AMP-activated protein kinase—fuel gauge of the mammalian cell?. Eur J Biochem.

[43] Shackelford DB, Shaw RJ (2009). The LKB1-AMPK pathway: metabolism and growth control in tumour suppression. Nat Rev Cancer.

[44] Gwinn DM, Shackelford DB, Egan DF, Mihaylova MM, Mery A, Vasquez DS, Turk BE, Shaw RJ (2008). AMPK phosphorylation of raptor mediates a metabolic checkpoint. Mol Cell.

[45] Shaw RJ (2009). LKB1 and AMP-activated protein kinase control of mTOR signalling and growth. Acta Physiol (Oxf).

[46] Hardie DG (2004). The AMP-activated protein kinase pathway—new players upstream and downstream. J Cell Sci.

[47] Green AS, Chapuis N, Lacombe C, Mayeux P, Bouscary D, Tamburini J (2011). LKB1/AMPK/mTOR signaling pathway in hematological malignancies: from metabolism to cancer cell biology. Cell Cycle.

[48] Menon S, Manning BD (2008). Common corruption of the mTOR signaling network in human tumors. Oncogene.

[49] Beelen K, Opdam M, Severson TM, Koornstra RH, Vincent AD, Wesseling J, Muris JJ, Berns EM, Vermorken JB, van Diest PJ, Linn SC (2014). Phosphorylated p-70S6K predicts tamoxifen resistance in postmenopausal breast cancer patients randomized between adjuvant tamoxifen versus no systemic treatment. Breast Cancer Res.

[50] Zakikhani M, Dowling R, Fantus IG, Sonenberg N, Pollak M (2006). Metformin is an AMP kinase-dependent growth inhibitor for breast cancer cells. Cancer Res.

[51] Keyomarsi K, Tucker SL, Buchholz TA, Callister M, Ding Y, Hortobagyi GN, Bedrosian I, Knickerbocker C, Toyofuku W, Lowe M, Herliczek TW, Bacus SS (2002). Cyclin E and survival in patients with breast cancer. N Engl J Med.

[52] Mittendorf EA, Liu Y, Tucker SL, McKenzie T, Qiao N, Akli S, Biernacka A, Liu Y, Meijer L, Keyomarsi K, Hunt KK (2010). A novel interaction between HER2/neu and cyclin E in breast cancer. Oncogene.

[53] Wang HX, Qin C, Han FY, Wang XH, Li N (2014). HIF-2α as a prognostic marker for breast cancer progression and patient survival. Genet Mol Res.

[54] Gilkes DM, Semenza GL (2013). Role of hypoxia-inducible factors in breast cancer metastasis. Future Oncol.

[55] Vaz-Luis I, Ottesen RA, Hughes ME, Marcom PK, Moy B, Rugo HS, Theriault RL, Wilson J, Niland JC, Weeks JC, Lin NU (2012). Impact of hormone receptor status on patterns of recurrence and clinical outcomes among patients with human epidermal growth factor-2-positive breast cancer in the National Comprehensive Cancer Network: a prospective cohort study. Breast Cancer Res.

[56] Kennecke H, Yerushalmi R, Woods R, Cheang MC, Voduc D, Speers CH, Nielsen TO, Gelmon K (2010). Metastatic behavior of breast cancer subtypes. J Clin Oncol.

[57] Paluch-Shimon S, Ben-Baruch N, Wolf I, Zach L, Kopolovic J, Kruglikova A, Modiano T, Yosepovich A, Catane R, Kaufman B (2009). Hormone receptor expression is associated with a unique pattern of metastatic spread and increased survival among HER2-overexpressing breast cancer patients. Am J Clin Oncol.

[58] Vaz-Luis I, Winer EP, Lin NU (2013). Human epidermal growth factor receptor-2-positive breast cancer: does estrogen receptor status define two distinct subtypes?. Ann Oncol.

[59] Bonotto M, Gerratana L, Iacono D, Minisini AM, Rihawi K, Fasola G, Puglisi F (2015). Treatment of Metastatic Breast Cancer in a Real-World Scenario: Is Progression-Free Survival With First Line Predictive of Benefit From Second and Later Lines?. Oncologist.

[60] Gong Y, Han EY, Guo M, Pusztai L, Sneige N (2011). Stability of estrogen receptor status in breast carcinoma: a comparison between primary and metastatic tumors with regard to disease course and intervening systemic therapy. Cancer.

[61] Lindström LS, Karlsson E, Wilking UM, Johansson U, Hartman J, Lidbrink EK, Hatschek T, Skoog L, Bergh J (2012). Clinically used breast cancer markers such as estrogen receptor, progesterone receptor, and human epidermal growth factor receptor 2 are unstable throughout tumor progression. J Clin Oncol.

